# Diagnostic use of neuro-spect quantified with ^99m^Tc-ECD a model based on normal data

**DOI:** 10.1590/S1980-57642013DN74000010

**Published:** 2013

**Authors:** Belmonte Juarez Marroni, Sabine Possa Marroni, Caroline Muller Mayer, Betina R. Braga

**Affiliations:** 1 Head of Nuclear Medicine Department, Hospital Ernesto Dornelles, Porto Alegre RS, Brazil.; 2 Neuropsychologist, Nuclimagem, Porto Alegre RS, Brazil.; 3 Nuclear Physician, Nuclimagem, Porto Alegre RS, Brazil.; 4 Medical Physicist, Nuclimagem. Porto Alegre RS, Brazil.

**Keywords:** SPECT, radiopharmaceutical, database, anatomical and functional normalization

## Abstract

**OBJECTIVE:**

The purpose of the present study was to prospectively quantify the normal limits of brain perfusion using specific software in a group of asymptomatic young volunteers submitted to single-photon emission tomography (SPECT) with ^99m^Tc-ethyl cysteinate dimer (^99m^Tc-ECD).

**METHODS:**

We used 15 MBq/kg of intravenous ^99m^Tc-ECD in 30 healthy volunteers submitted to brief neuropsychological tests and a clinical questionnaire. These data were normalized relative to the cerebellum (Group 1) and to the brain cortex (Group 2). Statistical analysis was performed with Student's t-test. The average radioactivity was 6.5 million counts/study.

**RESULTS:**

The normalized (Group I) revealed an average value of 78.03%, with standard deviation (SD) of 4.07. Two volunteers in this group had slightly greater than two standard deviations from the mean. When we used (Group II), the average value was 71.01%, with a SD of 4.66. We observed a difference between groups of 9% (P<0.001).

**CONCLUSION:**

The present study suggests normal cortical perfusional values for a group of young asymptomatic volunteers, utilizing ^99m^Tc-ECD. This allows further quantification of neuro-SPECT data, specifically comparisons across patient populations. Furthermore, this method represents a new tool that can be used to further explore the field of neuroimaging, particularly neuropsychiatry.

## Abstract (pt)

**OBJETIVO:**

O objetivo do presente estudo foi quantificar prospectivamente os limites normais de perfusão cerebral utilizando software específico em um grupo de jovens voluntários assintomáticos submetidos a tomografia por emissão de fóton Único (SPECT) com ^99m^Tc-etil cisteinato dímero (^99m^Tc-ECD).

**MÉTODOS:**

Foram utilizados 15 MBq/kg intravenoso de ^99m^Tc-ECD em 30 voluntários sadios submetidos a uma bateria breve de testes neuropsicológicos e questionário clínico. Estes dados foram normalizados para o cerebelo (Grupo 1) e no córtex cerebral (Grupo 2). A análise estatística foi obtida através do teste T de Student. A atividade média foi de 6,5 milhões de contagens/estudo.

**RESULTADOS:**

A normalização (Grupo I) revelou um valor médio de 78,03%, com um desvio-padrão (DP) de 4,07. Dois voluntários neste grupo tiveram valores ligeiramente maiores do que dois desvios padrões da média. Quando utilizado (Grupo II), o valor médio foi de 71,01%, com um desvio padrão de 4,66. Observou-se uma diferença entre grupos de 9% (P<0,001).

**CONCLUSÃO:**

O presente estudo sugere valores corticais perfusionais normais para um grupo de jovens voluntários assintomáticos, utilizando ^99m^Tc-ECD. Isto permite ainda a quantificação dos dados de neuro-SPECT, especificamente a comparação entre populações de pacientes. Além disso, este método apresenta uma nova ferramenta que pode ser usada para explorar ainda mais o campo da neuroimagem, particularmente em neuropsiquiatria.

## INTRODUCTION

Many previous studies have described the usefulness of scintigraphy cerebral perfusion, single-photon emission computed tomography (SPECT), as a qualitative/quantitative measure using hexamethyl-phenylethyleneaminexime (HMPAO) for neurological and psychiatric diseases.^1,2^ This investigative technique requires the use of protocols for data acquisition and analysis programs that contain basic population data. This technique also recognizes some differences in the biodistribution of the various neuroanatomical radiotracers.^3^ We developed a database using quantified ^99m^Tc ethyl cysteinate dimer (^99m^Tc-ECD) that included information about the normal variation of regional brain perfusion in asymptomatic volunteers that we believe will contribute to the development of the neuropsychiatry specialty. The database was developed using Mirage station and NeuroGam software on the Segami platform.

The gold standard for studying regional brain blood flow is the use of radiolabeled markers that cross the intact blood-brain barrier, typically the inert gas ^133^ Xe.^4,5^ For over two decades, molecular markers called static tracers, such as N-isopropyl-p-Iodoanphfetamine (IMP), HMPAO and ^99m^Tc ECD, have been used to study changes in relative regional blood flow using SPECT systems.^6^ This technique is called brain perfusion, and it relies on the coupled behavior of blood flow and metabolism, which is used as a marker of neuronal activity.^3,7^ Because of their chemical stability, biodistribution and facilitating biochemical properties, such as being neutral and lipophilic,^8^ these tracers are taken from brain circulation and are retained in neurons for many hours. The coefficient of extraction of these tracers is dependent on the neuronal metabolic state and on the vascular integrity.

The purpose of the present study was to assess and visualize the brain perfusion with ^99m^Tc- ECD using a quantification program with anatomical and functional standardization to allow the conducting of comparative analyses between populations and individuals with a new critical overview about brain dysfunction.

## METHODS

The present study aimed to evaluate normal brain perfusion with scintigraphy using ^99m^Tc-ECD from the Instituto de Pesquisas Energéticas e Nucleares (IPEN) in an asymptomatic group of volunteers and was approved by the research ethics committee of Hospital Ernesto Dornelles of Porto Alegre. The sample consisted of 30 volunteers (12 men (40%) and 18 women (60%)) aged between 18 and 30 years, with a mean age of 23.17 years (±3.45SD). This group was referred to as the Standard Group of Young Adult (SGYA). Among the subjects, there was a predominance of medium and high levels of education (mean 12.13 years ±2.76SD), which instilled increased confidence in the reliability of the answers given for the clinical questionnaire.

The data collection phase of the present study lasted for 45 days, during which all of the volunteers underwent a brief neuropsychological screening. The volunteers completed a standard informed consent form and answered a clinical questionnaire that was used to exclude individuals with a history of neurological, psychiatric, oncological or vascular disease, diabetes, traumatic brain injury, a history of cranial surgery, hypertension, epilepsy and individuals in chronic use of medications or drugs.

A brief neuropsychological screening was performed with the investigation prerequisite, aiming to exclude cognitive deficits, behavioral deficits and neuropsychiatric diseases, although without a depression scale. Several tasks were selected such as: sensitive neuropsychological executive function/attention and working memory tests / subtests. The Wechsler Adult Intelligence Scale (WAIS-R) subtest (digit span and digit symbol), phonemic Verbal Fluency (FAS), Attention/Concentration Test (AC) and Stroop test were administered to all participants.

For the scintigraphic procedure, the participants were required to abstain from the use of central nervous system (CNS) stimulants or depressants, such as alcohol, tobacco, xanthines, caffeine and psychotropic drugs, for at least 12 hours before the start of the investigation. Also, all volunteers that had a history of psychotrophic drugs use were excluded from the study.

The present study was performed with an IV injection of 15 MBq/kg of ^99m^Tc-ECD in an environment protected from sensory stimulation. The patients had their eyes closed and were placed in an environment without auditory or visual stress for five minutes before the injection. All of the doses were analyzed using column chromatography, with a minimum acceptance criterion of 95%. The acquisition of the images started between 30 and 60 minutes after the IV injection.

The data acquisition protocol used the following parameters: a window energy of 20% for ^99m^Tc with a 128 x 128 matrix, a low-energy high resolution (LEHR) collimator at 30 seconds per projection and 120 images in a Helix gamma camera (Elscint). The processing was performed using a ramp filter, and reconstruction was performed by filtered back projection. A Chang attenuation correction of 0.12 cm^-1^ was applied, and the data was post-filtered using a Butterworth filter (order 9, cutoff of 0.32 cycles/cm).

The data acquisition was performed on an eNTEGRA workstation from General Electric (GE). For quantification, the data were exported using NeuroGam software in a DICOM format to a Mirage workstation for the analysis of the brain perfusion.

During the data acquisition process, the traditional images were adjusted to a Talairach map and underwent a step known as anatomic standardization. The choice of the cerebellum as an anatomical landmark has both anatomical and functional advantages. This brain region is easily identifiable, is well defined and has an excellent perfusion status compared to other brain structures. Additionally, according to the work by Tumeh et al.,^9^ cerebellar metabolism has no direct relationship with age, and this region's metabolic loss is minimal during life.

Functional normalization is usually performed using the cerebellum. However, other brain regions can also be used. In the present study, the SGYA data that were normalized using the cerebellum were identified as Group I, and the SGYA data that were normalized relative to the cerebral cortex were identified as Group II. For this comparative analysis, we chose the bilateral lobes of the brain (frontal, temporal, parietal and occipital) and the cerebellum, thalamus, putamen and caudate points. All of the data were expressed as a percentage of the maximum voxel perfusion in relation to that of the cerebellum (Group I) or of the cerebral cortex (Group II). Subsequently, these data were compared using Student's t-test to characterize their similarities and differences. To determine the average global cortical perfusion level, we used all of Brodmann's areas in both hemispheres in addition to a joint analysis of each region. As an additional reference analysis, we chose Brodmann's area 44 (Broca's area). We used the z-score value, which is the value that is obtained by subtracting the average perfusion of the patient by average perfusion of the group divided by the standard deviation of the same group, to better identify the perfusional variables that were above or below 2 SD from the mean for each Brodmann's area.

**Measurement model.** This model was designed using the mathematical concept of volume in digital space and isotropic voxels.^8^ The unit of this division was the voxel, and the compartments that were formed were not the brain lobes, as is traditionally defined anatomically, but were spaces that were defined using a system of coordinates. By convention, we defined a reference line, denominated as the anterior and posterior commissures (AC-PC ), to be the line that follows the direction orbitomeatal direction.

These commissural references encompass each vertical line automatically and characterize a slice of each brain hemisphere into six compartments.

**Figure 1:**
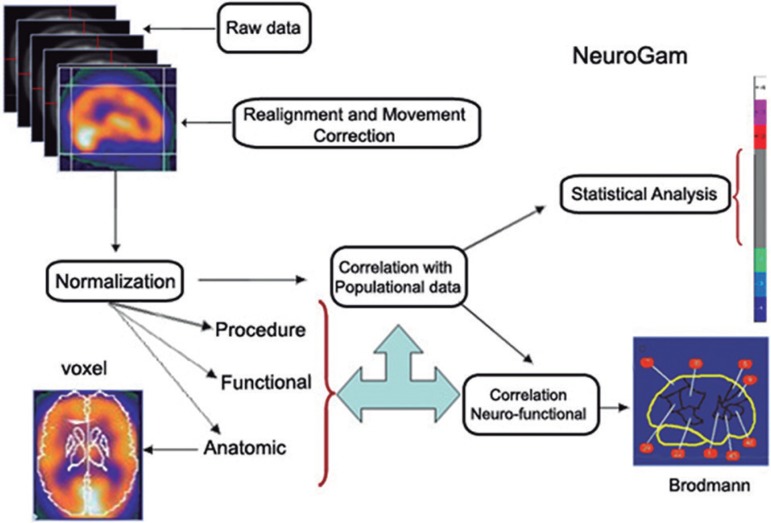
Schematic model of the acquisition and standardization of information.

**Processing of information.** These images underwent typical processing methods, which were used to eliminate noise - and movement-related artifacts, as shown in Figure 1.

Anatomic standardization allows for the localization of the radiotracer signal from different scans into the same mathematical space that is defined by three orthogonal planes. The user must always aim at the boundary of the brain cortex, with lines that are defined in the anatomical model of Talairach (10). Notably, the only large central nervous system (CNS ) structure that remains outside of the lines is the cerebellum.

The functional normalization step is based on a model of the maximum or average radioisotopic activity of a region of the brain. This technique is referred to as relative quantification, and it compares the reference to a known activity.

The model for relative quantification that is described in the present paper allows for a variety of information. The system allows the user to distinguish the brain perfusion in the three orthogonal planes, to distinguish the vascular area, to view Brodmann's areas and to view anatomical structures as a reference, such as the basal ganglia and the brain lobes.

In this way, the system uses AC-PC lines and provides volumes with finite areas, which is essential for comparative and serial analyses. The data for a particular selection are expressed as the number of pixels, its percentage in relation to the total area selected, and its standard deviation.

All of this information uses the reference that is chosen by the operator for the functional normalization from the cortical and subcortical structures. The reference that is chosen is usually the cerebellum for many reasons, including this region's greater relative activity, symmetry and high level of anatomical individualization. Moreover, this model allows for the selection of other references, including the entire brain, with average or maximum activity. We have considered the possibility of using the z-score, which yields a 99% inclusion of the SGYA data for each Brodmann's area. The result of this procedure is a display of the neuronal activity of a particular region, which is expressed using a color scale.

In the quantified model, the color scale acquires the connotation of a statistical expression. An analysis of this display data should be observed with caution because its statistical power is limited and is dependent on the incorporation of the population data to reliably express the main characteristics of the community that is being studied. Moreover, the comparison groups must have similar demographics, such as age, to be considered valid. Furthermore, for the comparisons to be appropriate there should be a greater similarity in the demographics between the patients under study and the normal controls.

**Figure 2:**
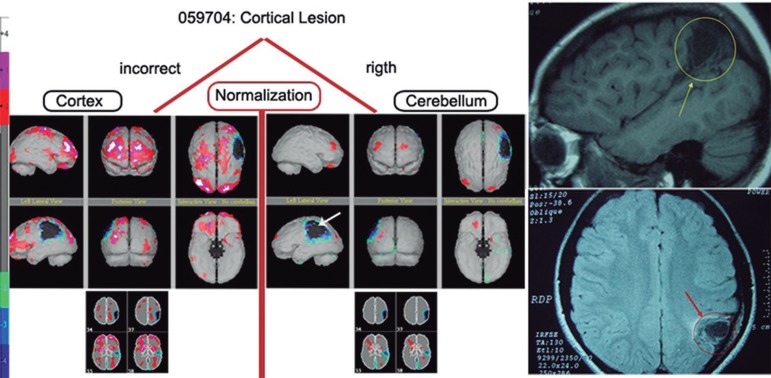
MRI and normalization types of case 1.

*Functional normalization: the importance of a correct reference –* Case 1: Patient CMFF is an 18-year-old female with a history of brain abscess in childhood who is currently diagnosed with attention deficit and learning difficulties. The brain perfusion demonstrated a significant cortical loss that was restricted to the peripheral area of the left parietal lobe, with no other differences in regional perfusion. This result was confirmed by magnetic resonance imaging, as shown in Figure 2. Below, we describe how to properly perform functional normalization and how to avoid hyperperfusion statistical artifacts using the cerebellum as a reference.

Conversely, when there is a cerebellar lesion and the cerebellum is used as a reference, there will be an artificially high level of cortical activity that prevents the proper interpretation of the regional brain perfusion. This result can lead to false positives and false negatives after the inappropriate use of functional normalization and its validation as a statistical tool.

**Figure 3:**
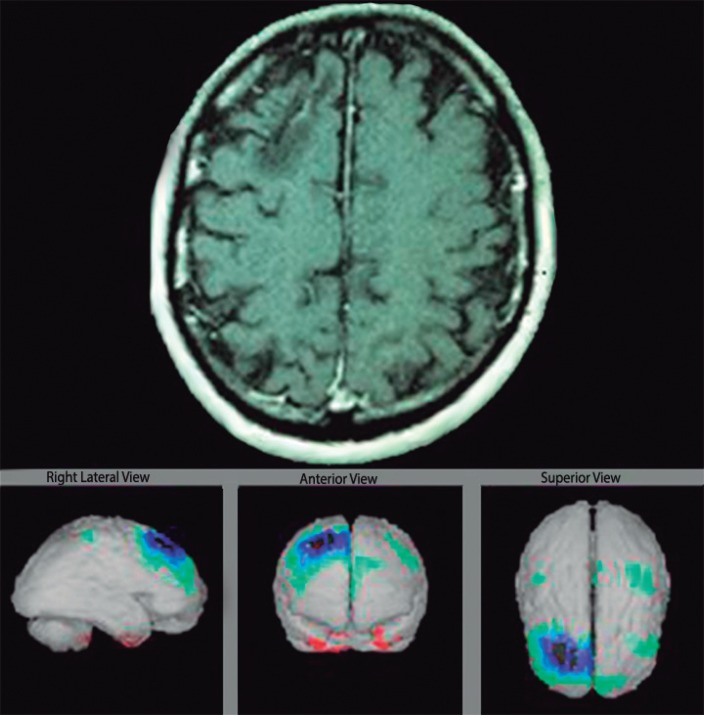
MRI and brain perfusion quantification of case 2.

Case 2: Patient DCL is a 47-year-old male with AIDS-positive serum, and an abnormal finding at the right frontal lobe from Magnetic Resonance Imaging (MRI) and history of headache, memory dysfunction, depression and treated for convulsion ictus for 13 years (Figure 3).

## RESULTS

All of the volunteers answered a clinical questionnaire that assessed their medical history, in order to exclude history of neuropsychiatric disorders. A brief neuropsychological screening was performed to exclude possible cognitive deficits, mainly those involving executive function, attention and working memory (Table 1). The SGYA group had a chromatographic average of 97.5% of binding of ^99m^Tc-ECD, and the average count of the radioactivity per acquisition was 6.5 million counts.

**Table 1:** Neuropsychological testing of the SGYA group.

Routine cognitive screening for cerebral perfusion results of volunteers - group 18-30 years									
N	Name	Gender	Age(years)	Educationallevel (years ofschooling)	Digit SymbolScaled score(FC)	Digit SpanScaled score(FC)	FocusedAttention(FC)	StroopTest III(FC)	Verbal Fluency(F,A,S)(FC)
1	R.S.T.	M	19	11	8 (MI)	9 (M)	70% (MS)	19"59 (+0,3 SD)	30 (-1,0 SD)
2	A.J.D.A.	M	21	10	8 (MI)	6 (MI)	90% (S)	23"25 (-0,4 SD)	34 (-0,6 SD)
3	B.B.B.D.	M	19	11	8 (MI)	7 (MI)	85% (S)	19"87 (+0,3 SD)	41 (0 SD)
4	G.G.S.D.	M	21	11	8 (MI)	11 (M)	75% (MS)	20"75 (+0,1 SD)	30 (-1,0 SD)
5	C.S.A.	F	30	7	6 (MI)	6 (MI)	35% (MI)	24"69 (0 SD)	30 (-0,7 SD)
6	G.C.B.	F	21	13	9 (M)	11 (M)	85% (S)	14"37 (+1,3 SD)	33 (-1,0 SD)
7	G.S.J.	F	18	8	6 (MI)	6 (MI)	20% (MI)	26"56 (-1,0 SD)	26 (-1,0 SD)
8	V.S.S.	F	24	11	9 (M)	8 (MI)	85% (S)	20"30 (+0,2 SD)	34 (-0,6 SD)
9	V.M.A.	F	30	16	11(M)	7 (MI)	20% (MI)	15"53 (+1,0 SD)	54 (+0,8 SD)
10	M.V.D.M.	M	22	10	7 (MI)	8 (MI)	80% (MS)	24"94 (-0,7 SD)	30 (-1,0 SD)
11	M.F.A.	M	20	10	9 (M)	10 (M)	80% (MS)	21"69 (-0,1 SD)	38 (-0,2 SD)
12	R.P.B.	M	20	11	8 (MI)	8 (MI)	95% (S)	23"43 (-0,4 SD)	30 (-1,0 SD)
13	M.H.S.C.	M	20	11	9 (M)	7 (MI)	90% (S)	20"75 (+0,1 SD)	37 (-0,3 SD)
14	W.D.	M	22	14	8 (MI)	6 (MI)	60% (M)	25"93 (-0,9 SD)	41 (-0,3 SD)
15	T.R.N.	M	23	14	10 (M)	14 (MS)	80% (MS)	15"47 (+1,1 SD)	45 (0 SD)
16	J.C.O.N.	M	20	11	11 (M)	6 (MI)	85% (MS)	18"94 (+0,4 SD)	55 (+1,3 SD)
17	J.M.G.	M	19	11	11 (M)	8 (MI)	85% (S)	26"75 (-1,0 SD)	60 (+1,8 SD)
18	S.L.P.C.	F	24	15	8 (MI)	10 (M)	65% (MS)	16"97 (+0,8 SD)	46 (+0,1 SD)
19	R.K.N.	F	26	14	12 (MS)	12 (MS)	65% (MS)	16"81 (+0,8 SD)	47 (+0,2 SD)
20	R.M.G.	F	21	11	7 (MI)	6 (MI)	85% (MS)	27"02 (-1,0 SD)	36 (-0,4 SD)
21	L.J.L.	F	25	16	10 (M)	7 (MI)	70% (MS)	24"10 (-0,5 SD)	49 (+0,4 SD)
22	F.L.	F	24	16	10 (M)	11 (M)	40% (M)	21"06 (0 SD)	40 (-0,4 SD)
23	G.S.S.	F	25	10	10 (M)	10 (M)	50% (M)	24"94 (-0,7 SD)	30 (-1,0 SD)
24	S.S.F.B.	F	25	8	8 (MI)	6 (MI)	70% (MS)	25"46 (0,8 SD)	26 (-1,0 SD)
25	Q.L.S.	F	27	15	10 (M)	6 (MI)	75% (MS)	19"28 (-0,4 SD)	35 (-0,9 SD)
26	L.M.P.	F	21	14	11 (M)	6 (MI)	35% (MI)	23"03 (-0,3 SD)	33 (-1,0 SD)
27	J.V.G.	F	25	10	6 (MI)	6 (MI)	20% (MI)	26"50 (-1,0 SD)	32 (-0,8 SD)
28	A.C.R.	F	26	11	8 (MI)	10 (M)	35% (MI)	25"35 (-0,7 SD)	30 (-1,0 SD)
29	B.B.	F	27	18	10 (M)	8 (MI)	45% (M)	23"98 (+0,1 SD)	33 (-1,0 SD)
30	R.N.	F	30	16	8 (MI)	7 (MI)	20% (MI)	22"19 (+0,3 SD)	45 (0 SD)

M: average; MI: below average; S: higher; MS: above average; SD: standard deviation (to -1.0 SD is considered normal); Age (years) mean 23,17 SD 3,45; Educational level (years) mean 12,13 SD 2,76; 18 Women (60%) and 12 Men (40%); FC: final classification.

The quantitative representation of the regional neuronal perfusion activity, which is localized to the gray matter of the brain, is provided by a color scale that is based on the normalized average values of the SGYA scans. Our study with ^99m^Tc-ECD showed that for this radiotracer, the mean brain perfusion value was 74.67%, with a standard deviation of 3.31 in the SGYA group. Therefore, if a test measurement changes within 2 SD of this value (between 68.05% and 81.29%), the event can be classified as normal. Such normal results are represented with gray coloring in the three-dimensional images. For deviations above the average normal range, hyperperfusion occurs (represented by colors ranging from red to white), which represents neuronal hyperactivity. Conversely, hypoperfusion is represented by colors that range from light green to dark blue to black and suggest a progressive reduction in neuronal activity in the topography of the cortical cerebral gray matter.

When we selected the bilaterally-paired Brodmann's areas to represent the average brain perfusion of the SGYA group, we noted that a small number of neuro-functional segments were more than 2 SD away from the mean. We believe that this result is due to statistically small deviations from normal that represent a physiological variant. We also observed relative hyperperfusion in the primary visual cortex and relative hypoperfusion in the inferior-mesial and dorsal-lateral temporal lobes (hippocampi and parahippocampal gyri) and in the middle area of the cingulate gyrus.

When the cerebellum was used as a reference in the functional normalization step (Group I), we found an average perfusion value of 78.03%, with a standard deviation of 4.07. This group had two volunteers with variations that were greater than one standard deviation away from the mean. When the cerebral cortex was used as a reference (Group II), the mean perfusion was 71.01%, with a standard deviation of 4.66. We observed a 9% perfusion decrease between Groups I and II, when the functional reference was changed.

When we only analyzed Brodmann's area 44 (Broca's area, which is responsible for implementation of language) in the 30 SGYA volunteers, we found that the relative quantification of the brain activity, with the cerebellum and cortex as references, reproduced the values shown above. The results of this analysis also confirmed the 9% variation between groups of relative perfusion when the reference was switched from the cerebellum to the cerebral cortex (Table 2 ).We observed a greater level of uptake in the occipital lobe and a lower level of uptake in the basal ganglia in both groups. This difference may have been related to the physiologic distribution of ^99m^Tc-ECD and also due to the physical characteristics of the image.

**Table 2:** Selective uptake between organs and groups.

Areas	Groups		
	U4 Cerebellum	U6 EB*	Percentage difference
Caudate Nucleus	55.92	50.98	11.62
Cerebellum	75.71	68.53	8.59
Cerebral Cortex	75.72	68.80	8.58
Frontal Lobe	72.84	66.20	8.92
Occipital Lobe	81.12	73.65	8.01
Parietal Lobe	74.34	67.58	8.74
Putamen	75.12	68.33	8.65
Temporal Lobe	72.31	65.54	8.99
Thalamus	67.88	62.46	9.58
Average of whole areas	72.33	65.79	-
Difference between groups	-	-	9.08

* EB: entire brain.

## DISCUSSION

Brain SPECT has been used in the field of nuclear medicine for diagnosis, prognosis and risk stratification. This scintigraphic technique has been shown to be important for vascular / neurodegenerative / psychiatric diseases, epilepsy, head trauma, malignant tumors, movement disorders, and drug addiction, as reported by Camargo and Heuser et al.^11-13^ The normal distribution of ^99m^Tc-ECD radiotracer in the brain is symmetric, with higher levels of activity in the occipital region and lower levels of activity in the temporal lobe and the posterior portion of the cingulate gyrus.^3^ The physical characteristics of ^99m^Tc-ECD are such that it is both neutral and lipophilic, which enables it to cross the blood-brain barrier and allows for the analysis of brain activity. The latter effect is possible because of this tracer's high intraneuronal concentration in gray matter compared to that in white matter.^2,14^ Furthermore, this tracer's brain blood flow is similar to the biodistribution of the gas ^133^Xe, which is between 20 and 80 ml/min/100g of brain tissue.^8^ The concentration and removal of ^99m^Tc-ECD from brain tissue depends on several factors. These factors include the intracellular metabolic state, specifically the ability of the neuron to maintain ^99m^Tc-ECD retention, the neural population density, blood supply, as well as the presence and concentration of esterases in the cell membrane.^15,18^ Many studies have demonstrated the differences between HMPAO and ^99m^Tc-ECD, which are both markers of brain perfusion.^3,7,18,19^ With ^99m^Tc-ECD, the reports point to a greater concentration because of neuronal density at the occipital and parietal cortices.^3,18^ This method uses the quantification model that was used in the present work and also used by Darcourt et al.^14^ These authors demonstrated that the data analyzed using NeuroGam, as compared to the other systems, for slices analysis and traditional qualitative images of ^99m^Tc-ECD activity in the brain cortex, were better.

This software used data from the traditional SPECT images in axial cuts, and the volume in which it was projected was the standardized anatomical model of Talairach.^10^ For the process of functional normalization, the cerebellum is typically chosen as a reference. The reasons for this choice are based on the topography of the body, which often differs between individuals and can differ from the standard anatomical model. Additionally, this region is chosen because of its high blood supply, neuronal density and neuro-functional stability compared to other structures.^18^ In cases of cerebellar disease, it is important to consider using another reference.

A quantified assessment of the functional normalization by the cerebellum (Group I) revealed an average perfusion value of 78.03%, with a standard deviation of 4.07. Two volunteers from this group had average values that were slightly greater than one standard deviation away from the mean. When the cortex was used as a reference (Group II), the average perfusion value was 71.01%, with a standard deviation of 4.66. Thus, switching the functional reference decreased the average perfusion between groups by 9%, with a 95% confidence interval of 7-11%. When considering only Brodmann's area 44, we confirmed this difference of 9%, which when comparing between groups, was statistically significant (p<0.001). Therefore, one may obtain inaccurate results when analyzing the neuro-functionality of this segment. As a result, the evaluation of brain perfusion indices with relative quantifications and z-score measures should be accompanied by additional studies, such as neuroanatomical Computed Tomography (CT) or MRI.

More recently,^18^ F-FDG studies were compared with SPECT studies for the early diagnosis of Alzheimer's disease.^15^ Both techniques used isotropic model voxels, a normal database, anatomic normalization, data recording and statistical tests. The results of these two techniques were similar. These techniques can now serve as important predictors of the development of a mild cognitive decline by identifying the areas of hypometabolism in the precuneus, posterior cingulate and parietal cortex.^15,20^ In conclusion, the present study suggests normal cortical perfusional values for a group of young asymptomatic volunteers, utilizing ^99m^Tc-ECD with the proposed standard clinical protocol.

It is evident that further quantified neuro-SPECT studies with a population-specific database and suitable tools for the analysis of correlations will provide an improved understanding of the behavior of neuro-functional structures and the brain. This technique will help lead the way towards a new frontier in contemporary neuropsychiatry and neuroimaging, both of which are yet to be explored.^21-23^

We also believe that the little that we have learned in this area of neuroscience points to a large variability in the dominance of brain regions and in the plasticity of neuronal functions. Therefore, it may be inappropriate to assume that anatomical and functional similarities exist among individuals in various higher cognitive functions, such as judgment, memory, language, calculation, orientation and behavior.

## References

[1] Migneco O, Darcourt J, Benoliel J (1994). Computerized localization of brain structures in single photon
emission computed tomography using a proportional anatomical stereotactic
atlas. Comput Med Imaging Graph.

[2] Morano GN, Seibyl JP (2003). Technical overview of brain SPECT imaging: improving acquisition
and processing of data. J Nucl Med Technol.

[3] Koyama M, Kawashima R, Ito H (1997). SPECT imaging of normal subjects with technetium-99m-HMPAO and
technetium-^99m-^ ECD. J Nucl Med..

[4] Obrist WD, Thompson HK, King CH, Wang HS (1967). Determination of regional cerebral blood flow by inhalation of
133-xenon. Circ Res.

[5] Celsis P, Goldman T, Henriksen L, Lassen NA (1981). A method for calculating regional cerebral blood flow from
emission computerized tomography of inert gas concentrations. J Comput Assist Tomogr.

[6] Holman BL, Helmann RS, Goldsmith SJ (1989). Biodistribution, dosimetry, and clinical evaluation of
technetium-99m ethyl cysteinate dimer in normal subjects and in patients
with chronic cerebral infarction. J Nucl Med.

[7] Jacquier-Sarlin MR, Polla BS, Slosman DO (1996). Cellular basis of ECD brain retention. J Nucl Med.

[8] Devous MD, Van Heertum RL, Tikofsky RS (2009). SPECT instrumentation, radiopharmaceuticals and technical
factors: functional cerebral SPECT and PET imaging. Functional Cerebral SPECT and PET Imaging.

[9] Tumeh PC, Alavi A, Houseni M (2007). Structural and functional imaging correlates for age-related
changes in the brain. Semin Nucl Med.

[10] Talairach J, Tournoux P (1988). Co-planar Stereotactic Atlas of the Human Brain.

[11] Camargo EE (2001). Brain SPECT in neurology and psychiatry. J Nucl Med.

[12] Heuser G, Mena I (1998). Neurospect in neurotoxic chemical exposure demonstration of
long-term functional abnormalities. Toxicol Ind Health.

[13] Miller BL, Ikonte C, Ponton M (1997). A study of the Lund-Manchester research criteria for
frontotemporal dementia: clinical and single-photon emission CT
correlations. Neurology.

[14] Darcourt J, Koulibaly PM, Migneco O (2002). Exploring the central nervous system state of the art
methodology. Médecine nucléaire.

[15] Minoshima S, Frei KA, Koeppe RA, Foster NL, Kuhl DE (1995). A diagnostic approach in Alzheimer's disease using
three-dimensional stereotactic surface projections of fluorine-18-FDG
PET. J Nucl Med.

[16] Papazyan JP, Delavelle J, Burkhard P (1997). Discrepancies between HMPAO and ECD SPECT imaging in brain
tumors. J Nucl Med.

[17] Schiepers C, Verbruggen A, Casaer P, De Roo M (1997). Normal brain perfusion pattern of technetium-99m ethilcysteinate
dimer in children. J Nucl Med.

[18] Oku N, Matsumoto M, Hashikawa K (1997). Intra-individual differences between technetium-99m-HMPAO and
technetium-^99m^-ECD in the normal medial temporal
lobe. J Nucl Med.

[19] Mena FJ, Mena I, Alamos F (1998). Children normal HMPAO brain SPECT. Alasbimn Journal.

[20] Matsuda H (2007). Role of neuroimaging in Alzheimer's disease, with emphasis on
brain perfusion SPECT. J Nucl Med.

[21] Mena I, Correa R, Nader A (2004). Bipolar affective disorders: assessment of functional brain
changes by means of Tc99m HMPAO NeuroSPECT. Alasbimn Journal.

[22] Mena I, Prado C, Correa M (2001). Comparative functional study of two psychiatric pathologies by
means of BrainSPECT Tc99 HMPAO. Major depression and borderline personality
disorder. Alasbimn Journal.

[23] Mena I (2009). Neurospect applications in psychiatry. Alasbimn Journal.

